# Supplementary material for: De novo design of modular peptide-binding proteins by superhelical matching
Source: Nature. 2023 Apr 5;616(7957):581–9. doi: 10.1038/s41586-023-05909-9 (PMC10115654; doi:10.1038/s41586-023-05909-9)

nature portfolio

https://doi.org/10.1038/s41586-023-05909-9

Supplementary information

De novo design of modular peptide-binding proteins by superhelical matching

In the format provided by the authors and unedited

RAW DATA (Western Blots)

Figure 6E

Anti-ZFC3H1

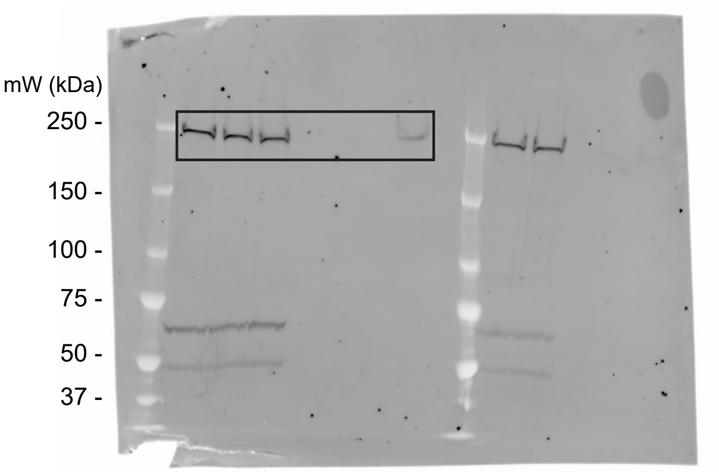

Anti-Tubulin 488

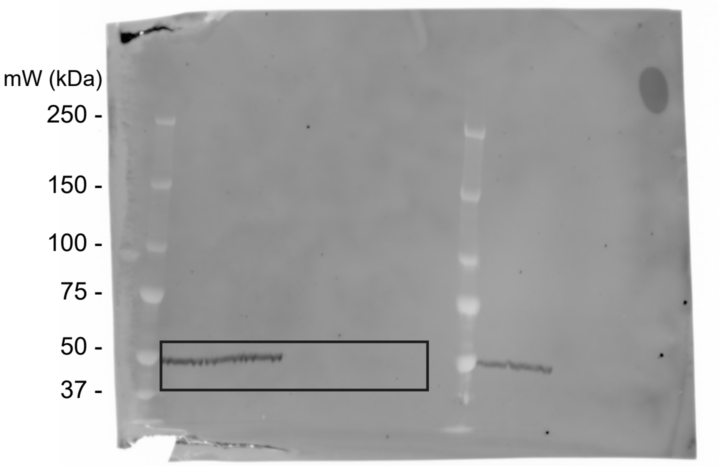

Coomassie stained gel

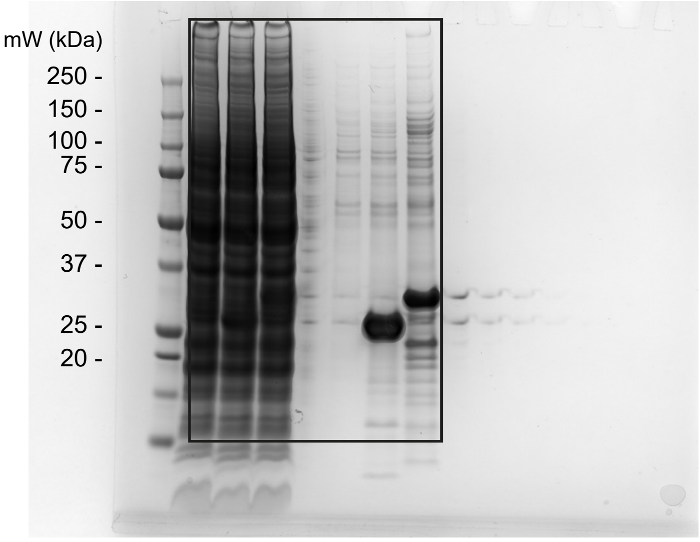

Extended Data Fig.12B

Anti-ZFC3H1

Experiment #3 Experiment #2

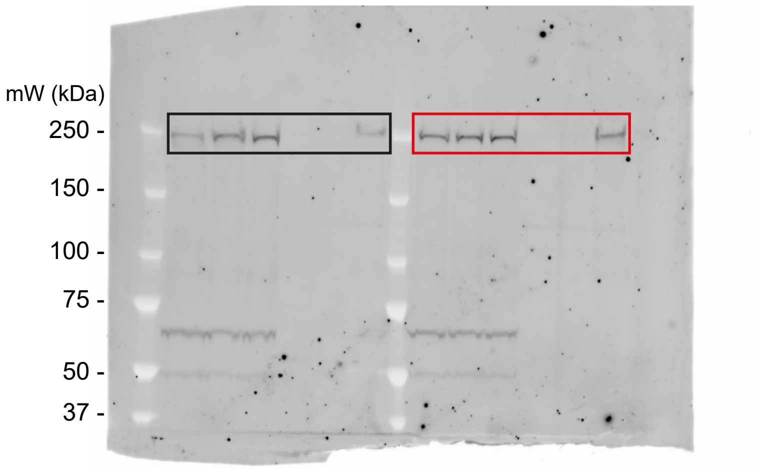

Anti-Tubulin 488

Experiment #3 Experiment #2

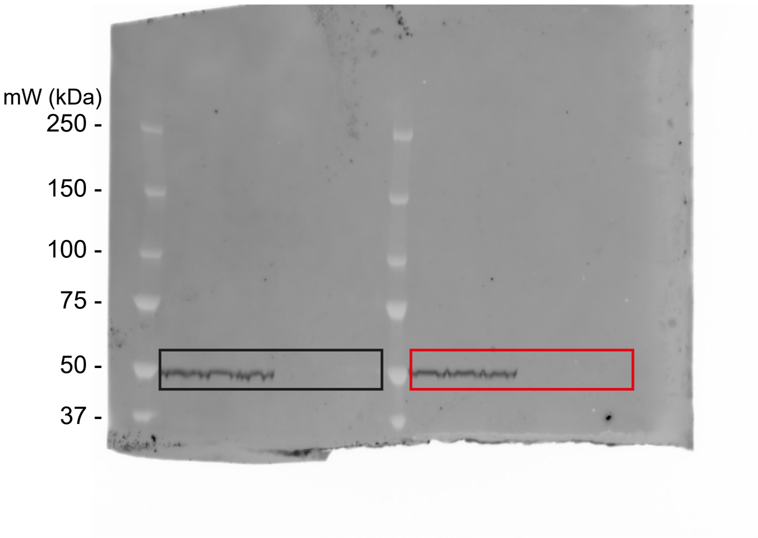

Supplement: Supplementary file 1 — This file contains the raw data (western blots) for Fig. 6 and Extended Data Fig. 7. [file 41586_2023_5909_MOESM1_ESM.pdf]
