# Supplementary material for: De novo design of modular peptide-binding proteins by superhelical matching
Source: Nature. 2023 Apr 5;616(7957):581–9. doi: 10.1038/s41586-023-05909-9 (PMC10115654; doi:10.1038/s41586-023-05909-9)
Supplement: Supplementary file 2 — Reporting Summary [file 41586_2023_5909_MOESM2_ESM.pdf]

# nature portfolio

Corresponding author(s): David Baker, Daniel Adriano Silva, Emmanuel Derivery

Last updated by author(s): Dec 8, 2022

# Reporting Summary

Nature Portfolio wishes to improve the reproducibility of the work that we publish. This form provides structure for consistency and transparency in reporting. For further information on Nature Portfolio policies, see our Editorial Policies and the Editorial Policy Checklist.

## Statistics

For all statistical analyses, confirm that the following items are present in the figure legend, table legend, main text, or Methods section.

| n/a | Confirmed | |
|---|---|---|
| ☐ | ☒ | The exact sample size (*n*) for each experimental group/condition, given as a discrete number and unit of measurement |
| ☐ | ☒ | A statement on whether measurements were taken from distinct samples or whether the same sample was measured repeatedly |
| ☒ | ☐ | The statistical test(s) used AND whether they are one- or two-sided<br>*Only common tests should be described solely by name; describe more complex techniques in the Methods section.* |
| ☐ | ☒ | A description of all covariates tested |
| ☐ | ☒ | A description of any assumptions or corrections, such as tests of normality and adjustment for multiple comparisons |
| ☐ | ☒ | A full description of the statistical parameters including central tendency (e.g. means) or other basic estimates (e.g. regression coefficient) AND variation (e.g. standard deviation) or associated estimates of uncertainty (e.g. confidence intervals) |
| ☒ | ☐ | For null hypothesis testing, the test statistic (e.g. *F*, *t*, *r*) with confidence intervals, effect sizes, degrees of freedom and *P* value noted<br>*Give P values as exact values whenever suitable.* |
| ☒ | ☐ | For Bayesian analysis, information on the choice of priors and Markov chain Monte Carlo settings |
| ☒ | ☐ | For hierarchical and complex designs, identification of the appropriate level for tests and full reporting of outcomes |
| ☒ | ☐ | Estimates of effect sizes (e.g. Cohen's *d*, Pearson's *r*), indicating how they were calculated |

*Our web collection on statistics for biologists contains articles on many of the points above.*

## Software and code

Policy information about availability of computer code

| | |
|---|---|
| Data collection | Microscopy data was collected using Metamorph software v7.10.1.161.<br>Rosetta Macromolecular Modeling Suit; |
| Data analysis | Image analysis was performed using Fiji (ImageJ version: 1.53f). Mass Spectrometry data was analyzed using Scaffold.<br>Python 3.8; ForteBio Data Analysis Software Version 9.0.0.14; FlowJo v10.6.2; Phenix-1.19.2; DNAWorks2.0. |

For manuscripts utilizing custom algorithms or software that are central to the research but not yet described in published literature, software must be made available to editors and reviewers. We strongly encourage code deposition in a community repository (e.g. GitHub). See the Nature Portfolio guidelines for submitting code & software for further information.

## Data

Policy information about availability of data

All manuscripts must include a data availability statement. This statement should provide the following information, where applicable:

- Accession codes, unique identifiers, or web links for publicly available datasets
- A description of any restrictions on data availability
- For clinical datasets or third party data, please ensure that the statement adheres to our policy

The mass spectrometry proteomics has been deposited to the ProteomeXchange Consortium via the PRIDE partner repository with the dataset identifier PXD038492 and 10.6019/PXD038492. The atomic coordinates and experimental data of RPB_PEW3_R4-PAWx4, RPB_PLP3_R6-PLPx6, RPB_LRP2_R4-LRPx4,

RPB_PLP1_R6-PLPx6, RPB_PLP1_R6-PLPx6 (alternative conformation 1), RPB_PLP1_R6-PLPx6 (alternative conformation 2) and RPB_LRP2_R4 (pseudopolymeric) have been deposited in the RCSB PDB with the accession numbers 7UDJ, 7UE2, 7UDK, 7UDL, 7UDM, 7UDN, and 7UDO respectively.
All other data supporting the findings of this study are available from the corresponding authors on reasonable request.

## Human research participants

Policy information about studies involving human research participants and Sex and Gender in Research.

| | |
|---|---|
| Reporting on sex and gender | N/A |
| Population characteristics | N/A |
| Recruitment | N/A |
| Ethics oversight | N/A |

Note that full information on the approval of the study protocol must also be provided in the manuscript.

# Field-specific reporting

Please select the one below that is the best fit for your research. If you are not sure, read the appropriate sections before making your selection.

☒ Life sciences ☐ Behavioural & social sciences ☐ Ecological, evolutionary & environmental sciences

For a reference copy of the document with all sections, see nature.com/documents/nr-reporting-summary-flat.pdf

# Life sciences study design

All studies must disclose on these points even when the disclosure is negative.

| | |
|---|---|
| Sample size | 30-60 designs were ordered for each batch of experimental characterization. No statistical method was used to determine the total number. |
| Data exclusions | No data was excluded. |
| Replication | Experimental finders were statistically significant and no attempt at reproduction was performed. |
| Randomization | Randomization was not relevant/not performed. |
| Blinding | Researchers were not blinded/not necessarily blind to perform all the binding assays. |

# Reporting for specific materials, systems and methods

We require information from authors about some types of materials, experimental systems and methods used in many studies. Here, indicate whether each material, system or method listed is relevant to your study. If you are not sure if a list item applies to your research, read the appropriate section before selecting a response.

### Materials & experimental systems

| n/a | Involved in the study | |
|---|---|---|
| ☐ | ☒ | Antibodies |
| ☐ | ☒ | Eukaryotic cell lines |
| ☒ | ☐ | Palaeontology and archaeology |
| ☒ | ☐ | Animals and other organisms |
| ☒ | ☐ | Clinical data |
| ☒ | ☐ | Dual use research of concern |

### Methods

| n/a | Involved in the study | |
|---|---|---|
| ☒ | ☐ | ChIP-seq |
| ☐ | ☒ | Flow cytometry |
| ☒ | ☐ | MRI-based neuroimaging |

## Antibodies

| | |
|---|---|
| Antibodies used | TOM20 antibody (Santa Cruz sc-17764, used it at 1:200 dilution), combined with anti-mouse Alexa Fluor 488 (Invitrogen, A21202 1:500e) for immunofluorescence. Rabbit anti-ZFC3H1 (Sigma, HPA007151, used at 1:250) combined with goat anti-Rabbit Alexa 555 (Invitrogen, A32732, 1:2000) for western blot. Mouse anti-alpha tubulin 488 (Clone DMA1, Sigma T6199, directly labelled with Abberior® STAR 488, NHS ester leading to a 4.5 dye/antibody degree of labelling, and used at 0.1 µg/mL final concentration) for |

| | |
|---|---|
| | western blot. |
| Validation | Monoclonal DM1A antibody anti alpha tubulin has been characterized by the manufacturer (https://www.sigmaaldrich.com/GB/en/product/sigma/t6199) . TOM20 antibody has been characterized by the manufacturer (https://datasheets.scbt.com/sc-17764.pdf). |

## Eukaryotic cell lines

Policy information about cell lines and Sex and Gender in Research

| | |
|---|---|
| Cell line source(s) | U2OS FlipIn Trex Cells were a kind gift from Stephen C. Blacklow. HeLa FlpIn Trex cells were a kind gift of Simon Bullock |
| Authentication | Cell line used was not authenticated |
| Mycoplasma contamination | Dapi staining did not reveal the presence of Mycoplasms |
| Commonly misidentified lines (See ICLAC register) | No commonly misidentified lines were used |

## Flow Cytometry

### Plots

Confirm that:

- [x] The axis labels state the marker and fluorochrome used (e.g. CD4-FITC).
- [x] The axis scales are clearly visible. Include numbers along axes only for bottom left plot of group (a 'group' is an analysis of identical markers).
- [ ] All plots are contour plots with outliers or pseudocolor plots.
- [ ] A numerical value for number of cells or percentage (with statistics) is provided.

### Methodology

| | |
|---|---|
| Sample preparation | EBY100 yeast cells were used for yeast surface display and subjected to the flow cytometry assay. The expression of protein is labeled by FITC conjugated anti-cMyc antibody, while the target is biotinlyated and labeled by SAPE. The samples are prepared and washed in PBS with 1% BSA |
| Instrument | SONY SH800 sorter and Attune NxT Flow Cytometer |
| Software | Softwares of the instruments and python |
| Cell population abundance | The collected yeast cell populations will be grown up in CTUG culture again, and if there is contamination, the cells won't grown well. In most of case, we will also inoculate some liquid yeast cells onto CTUG plates, and check single colony samples to make sure the purify of the cell and check the single colony sequences. |
| Gating strategy | Usually, there will be a control sort, in which there are no target presented. The gating is determined by the control sort. |

- [ ] Tick this box to confirm that a figure exemplifying the gating strategy is provided in the Supplementary Information.
