# Supplementary material for: De novo design of modular peptide-binding proteins by superhelical matching
Source: Nature. 2023 Apr 5;616(7957):581–9. doi: 10.1038/s41586-023-05909-9 (PMC10115654; doi:10.1038/s41586-023-05909-9)
Supplement: Supplementary file 4 — Crystallographic data collection and refinement statistics. [file 41586_2023_5909_MOESM4_ESM.pdf]

Table 1 Data collection and refinement statistics

	RPB_PEW3_R4-PAWx4	RPB_PLP3_R6-PLPx6	RPB_LRP2_R4-LRPx4	RPB_PLP1_R6-PLPx6
**Data collection**				
Space group	$P2_12_12_1$	*I422*	$P3_221$	*R32*
Cell dimensions				
a, *b*, *c* (Å)	45.4, 67.6, 71.3	124.5, 124.5, 105.0	70.8, 70.8, 128.2	99.4, 99.4, 173.2
α, β, γ (°)	90.0, 90.0, 90.0	90.0, 90.0, 90.0	90.0, 90.0, 120.0	90.0, 90.0, 120.0
Beamline	APS 23-ID-D	APS 23-ID-D	APS 23-ID-B	APS 23-ID-B
Wavelength	1.033	1.033	1.033	1.033
Resolution (Å)	38.3 - 2.70 (2.80-2.70)*	45.1-2.68 (2.78-2.68)	35.4-3.18 (3.92-3.18)	37.7-2.15 (2.21-2.15)
Total Observations	39,005	155,310	64,884	186,166
Unique reflections	6,383	11,891	6,621	18,220
Redundancy	6.1 (6.4)	13.1 (13.3)	9.8 (10.2)	10.2 (10.5)
Completeness (%)	99.6 (99.8)	92.1 (77.4)	89.1 (67.9)	99.9 (100)
R_{meas}	0.08 (1.14)	0.11 (2.51)	0.08 (3.38)	0.06 (2.45)
$I/\sigma(I)$	12.0 (1.27)	18.5 (1.21)	14.1 (0.69)	18.1 (0.85)
$CC_{1/2}$	1.00 (0.81)	1.00 (0.51)	1.00 (0.69)	1.00 (0.45)
**Refinement**				
Resolution (Å)	38.3 - 2.70	45.1 - 2.68	35.4 - 3.18	37.7 - 2.15
No. reflections				
Work	6,057	10,412	5,357	15,249
Test	316	547	294	1,672
R_{work} / R_{free}	24.5 / 27.0	22.6 / 27.7	20.7 / 25.6	23.2 / 27.6
No. atoms				
Protein	1,502	2,375	1,372	2,172
Peptide	104	139	104	240
Water	0	0	0	7
Mean B-factor (Å^2)	114.9	86.9	162.9	86.9
R.m.s. deviations				
Bond lengths (Å)	0.002	0.002	0.004	0.002
Bond angles (°)	0.41	0.49	0.62	0.41
Ramachandran[1]				
Favored (%)	98.0	98.4	97.2	99.3
Outliers (%)	0.0	0.0	0.6	0.0
Rotamers[1]				
Favored (%)	96.20	98.8	94.08	97.1
Outliers (%)	0.0	0.0	0.0	0.0
MolProbity[1]				
MolProbity Score	1.10	1.38	1.78	1.01
Percentile	100th	100th	100th	100th
Clashscore	3.12	6.99	13.35	3.06
Percentile	100th	99th	95th	99th
PDB ID	7UDJ	7UE2	7UDK	7UDL

*Values in parentheses are for highest-resolution shell.

[1] As reported by MolProbity.

Table 1 Cont. Data collection and refinement statistics

	RPB_PLP1_R6-PLPx6, alt conf 1	RPB_PLP1_R6-PLPx6, alt conf 2	RPB_LRP2_R4, pseudopolymeric
**Data collection**			
Space group	$P22_12_1$	$P22_12_1$	$P3_221$
Cell dimensions			
a, *b*, *c* (Å)	54.4, 80.3, 154.4	80.4, 86.5, 110.2	72.6, 72.6, 127.3
α, β, γ (°)	90.0, 90.0, 90.0	90.0, 90.0, 90.0	90.0, 90.0, 120.0
Beamline	APS 23-ID-B	APS 23-ID-B	APS 23-ID-B
Wavelength	1.033	1.033	1.033
Resolution (Å)	43.2 - 2.65 (2.72-2.65)*	43.2 - 2.45 (2.51-2.45)	44.7 - 2.50 (2.57-2.50)
Total Observations	135,662	191,404	139,040
Unique reflections	20,350	28,936	13,974
Redundancy	6.7 (6.7)	6.6 (6.7)	9.9 (9.8)
Completeness (%)	99.8 (100.0)	99.9 (100.0)	100.0 (100.0)
R_{meas}	0.10 (2.11)	0.07 (1.85)	0.07 (2.18)
$I/\sigma(I)$	10.9 (0.9)	14.0 (1.2)	18.6 (1.0)
$CC_{1/2}$	1.00 (0.57)	1.00 (0.49)	1.00 (0.48)
**Refinement**			
Resolution (Å)	43.2 - 2.65	43.2 - 2.45	44.7 - 2.50
No. reflections			
Work	16,916	25,465	12,425
Test	1,167	1,466	659
R_{work} / R_{free}	26.0 / 29.9	23.5 / 26.7	19.3 / 21.9
No. atoms			
Protein	4,492	4,565	1,399
Peptide	88	0	0
Water	0	0	0
Mean B-factor (Å^2)	113.7	91.4	89.7
R.m.s. deviations			
Bond lengths (Å)	0.001	0.002	0.012
Bond angles (°)	0.294	0.339	1.353
Ramachandran[1]			
Favored (%)	98.9	99.1	98.2
Outliers (%)	0.0	0.0	0.0
Rotamers[1]			
Favored (%)	99.2	97.6	97.2
Outliers (%)	0.4	0.9	0.7
MolProbity[1]			
MolProbity Score	1.04	0.94	1.28
Percentile	100th	100th	100th
Clashscore	2.58	1.84	5.24
Percentile	100th	100th	99th
PDB ID	7UDM	7UDN	7UDO

*Values in parentheses are for highest-resolution shell.

[1] As reported by MolProbity.
