# Supplementary material for: De novo design of modular peptide-binding proteins by superhelical matching
Source: Nature. 2023 Apr 5;616(7957):581–9. doi: 10.1038/s41586-023-05909-9 (PMC10115654; doi:10.1038/s41586-023-05909-9)
Supplement: Supplementary file 5 — Amino acid sequences of the designed proteins and designed peptides provided in this work. [file 41586_2023_5909_MOESM5_ESM.pdf]

ProteinID	Protein Name	Peptide Target	Protein Sequence
RPB_PLP1_R6-PLPx6	R6PO11	PLPPLPPLPPLPPLPPLP	PEEERIKYVITVVEQIAKDAHRNGQEELAKLAERTAEEAKKATERGEEETLRIVYVIVVVLQIALEAHRNGQEELAKLALRTAEEAIKATERGEEETLRIVYVIVVVLQIALEAHRNGQEELAKLALRTAEEAIKATERGEEETLRIVYVIVVVLQIALEAHRNGQEELAKLALRTAEEAIKATERGEEETLRIVYVIVVVLQIALEAHRNGQEELAKLALRTAEEAIKATERGEEETERIVYDIVVVLQEALEAHRNGEEERAKKALDEARRRIEATERGE
RPB_PLP1_R4-PLPx4	R4PO11	PLPPLPPLPPLP	PLRIVYVIAVVLQIVKDAHRNGQEELAKLAQRTAEEAIKAIERGEEETLRIVYVIAVVLQIALDAHRNGQEELAKLALRTAEEAIKAIERGEEETLRIVYVIAVVLQIALDAHRNGQEELAKLALRTAEEAIKAIERGEEETERIVYDIAVVLQEALDAHRNGEEERAKKALDEARRRIEAIERGE
RPB_PLP2_R6-PLPx6	R6D2	PLPPLPPLPPLPPLPPLP	PEEERIKYVITVVEQIAKDAHRNGQEELAKLAERTAEEAKKAIERGEEETLRIVYVIAVVLQIALDAHRNGQEELAKLALRTAEEAIKAIERGEEETLRIVYVIAVVLQIALDAHRNGQEELAKLALRTAEEAIKAIERGEEETLRIVYVIAVVLQIALDAHRNGQEELAKLALRTAEEAIKAIERGEEETLRIVYVIAVVLQIALDAHRNGQEELAKLALRTAEEAIKAIERGEEETERIVYDIAVVLQEALDAHRNGEEERAKKALDEARRRIEAIERGE
RPB_PLP3_R6-PLPx6	R6PEP12	PLPPLPPLPPLPPLPPLP	DEEREKLKEKLKEVLRRAKEAKKKGDKEKLIELAYEAAALAAWIIHKDSNDDEIVELAKEALKLVLEAAKEAKKNGDKEKLIKLAYLAAAVAAWIIHTDGDDDEIVELAKEALKLVLEAAKEAKKNGDKEKLIKLAYLAAAVAAWIITTDGDDDEIVELAKEALKLVLEAAKEAKKNGDKEKLIKLAYLAAAVAAWIIHTDGDDDEIVELAKEALKLVLEAAKEAKKNGDKEKLIKLAYLAAAVAAWIITTDGDDEEIVELAKEALKLVKEAAEEAEKQGDEELREKLRYLSEAVREWIERND
RPB_LRP1_R6-LRPx6	R6ST3	LRPLRPLRPLRPLRPLRP	PEEEAHKKAMIKIDVAFDYGKREEELEKILEEAIKELEKNGVDKEEAAHRAAMILIDVAFDYGKDDKELEKILEIAIKILEKNGVDKEEAAHRAAMILIDVAFDYGKDDKELEKILEIAIKILEKNGVDKEEAAHRAAMILIDVAFDYGKDDKELEKILEIAIKILEKNGVDKEEAAHRAAMILIDVAFDYGKDDKELEKILEIAIKILEKNGVDKREAAKRAAMILRDVADDYGKHEEEEEKIEEIAKKILEKNGHE
RPB_LRP2_R6-LRPx6	R6PXX13	LRPLRPLRPLRPLRPLRP	PEEEAFLKASIRIQHAHEHGKREEELEKILEEAIRELEKNGVDREEAAFLAASILIQHAHEQGKDDRELEKILEIAIRILEKNGVDREEAAFLAASILIQHAHEQGKDDRELEKILEIAIRILEKNGVDREEAAFLAASILIQHAHEQGKDDRELEKILEIAIRILEKNGVDREEAAFLAASILIQHAHEQGKDDRELEKILEIAIRILEKNGVDRREAAFLAASILIQHAREQGKHEEEEERIEEIAKRILEKNGHE
RPB_LRP2_R4-LRPx4	R4PXX13	LRPLRPLRPLRP	PEEEAFLKASIRIQHAHEHGKREEELEKILEEAIRELEKNGVDREEAAFLAASILIQHAHEQGKDDRELEKILEIAIRILEKNGVDREEAAFLAASILIQHAHEQGKDDRELEKILEIAIRILEKNGVDRREAAFLAASILIQHAREQGKHEEEEERIEEIAKRILEKNGHE
RPB_LRP2_R6_FW6	R6PXX13M	LRPLRPLRPLRPLRPLRP	PEEEAWLKASIRIQSAHEHGKREEELEKILEEAIRELEKNGVDREEAAWLAASILIQSAHEQGKDDRELEKILEIAIRILEKNGVDREEAAWLAASILIQSAHEQGKDDRELEKILEIAIRILEKNGVDREEAAWLAASILIQSAHEQGKDDRELEKILEIAIRILEKNGVDRREAAWLAASILIQSAREQGKHEEEEERIEEIAKRILEKNGH
RPB_PEW1_R6-PAWx6	R6n11	PEWPEWPEWPEWPEWPEW	PEREKVEKLARNAHLWAKKKLEEAKRHGNEEETEEWKRETELVRRLAELAHKSDREEVLKVIKLALNALLWANKKLDEAKRHGNEEETEEWRKETELVKRLAELASKSDREEVLKVIKLALNALLWANKKLDEAKRHGNEEETEEWRKETELVKRLAELASKSDREEVLKVIKLALNALLWANKKLDEAKRHGNEEETEEWRKETELVKRLAELASKSDREEVLKVIKLALNALLWANKKLDEAKRHGNEEETEEWRKETELVKRLAELASKSDREEVLKTIKLALNNLLWANKKLDEAKRHGNEEETEHWRKRTETVKREAEKASKSDK
RPB_PEW2_R6-PAWx6	R604	PEWPEWPEWPEWPEWPEW	PEEKLKREVLQIARAAIYWQRNGGDEHVEKALKRVKRAAEELGNSEEEALKRLVLQIARAAIYWQRNGGDEYVEAALKVVKKAAEELGNSEEEALKRLVLQIARAAIYWQRNGGDEYVEAALKVVKKAAEELGNSEEEALKRLVLQIARAAIYWQRNGGDEYVEAALKVVKKAAEELGNSEEEALKRLVLQIARAAIYWQRNGGDEYVEAALKVVKKAAEELGNSEDEALKRLKLQIARAAHYWENNGGEEYVKAAEKVEKKAAEELGNE
RPB_PEW1_R4-PAWx4	R4n11	PEWPEWPEWPEW	PEVERLRKEVKEEADKARENNDEDSLRKWKLLANALIAAHFAHLNGLEEVARLATEVVHEAYEASRNNDEDSLRKWLLLANALIAAHFAHLNGLEEVARLATEVVHEAYEASRNNDEDSLRKWLLLANALIAAHFAHLNGLEEVAHEATRVVHEATRASEENDEESLEKWLLEANKLIATHFAHLNGE
RPB_PEW3_R4-PAWx4	R4PXX28	PEWPEWPEWPEW	PEEERKKEAEEVAAHVEQIAFIAKEQGNEEVAKLAKRLAETIKRLNEGTEEEVKRLLEAAEVAAHVLQIAFIAHEQGNEEVAKLALELAESILRLIEGTEEEVKRLLEAAEVAAHVLQIAFIAHEQGNEEVAKLALELAESILRLIEGTEEEVKELLERAEEAAHVLQHAFIATEQGNEEDAKEALRKAEEILRRNEDRR
RPB_IYP1_R6-IYPx6	R602	IYPIYPIYPIYPIYPIYP	PEEEILVKAVNEASRITEKTKDEEKIKEALERILREAAERIRENGGNEEEAILVIAVNVASRITERVKNEEEIKKALEWILRVAAELIRENGGNEEEAILVIAVNVASRITERVKNEEEIKKALEWILRVAAELIRENGGNEEEAILVIAVNVASRITERVKNEEEIKKALEWILRVAAELIRENGGNEEEAILVIAVNVASRITERVKNEEEIKKALEWILRVAAELIRENGGNEEEAILVIAVNVASRITKRVTNEEEIKKAQHWIKHVAKKLIKENGGT
	R615M	IYPIYPIYPIYPIYPIYP	PEEKLEREVLQIARAAIYWQRNGGDEHVEKALKRVKRAAEELGNSEEEALGVLVAQIAHMAIHWQRNGGDEYVEAALKVVKKAAEELGNSEEEALGVLVAQIAHMAIHWQRNGGDEYVEAALKVVKKAAEELGNSEEEALGVLVAQIAHMAIHWQRNGGDEYVEAALKVVKKAAEELGNSEEEALGVLVAQIAHMAIHWQRNGGDEYVEAALKVVKKAAEELGNSEDEALERLKLQIARAAHYWENNGGEEYVKAAEKVEKKAAEELGNE
RPB_IYP1_R4-IYPx4	R402	IYPIYPIYPIYP	PEEEILVKAVNEASRITEKTKDEEKIKEALERILREAAERIRENGGNEEEAILVIAVNVASRITERVKNEEEIKKALEWILRVAAELIRENGGNEEEAILVIAVNVASRITERVKNEEEIKKALEWILR

			VAAELIRENGGNEEEAILVIAVNVASRITKRVTNEEEIKKAQHWIKHVAKKLIKENGGT
RPB_PRM1_R6-PRMx6	R6CP33	PRMPRMPRMPRMPRMPRM	PEEKLYWEVSQIAFSAIEEQKNGGDEHVEKALKKVKKAAEELGNSEEEALYWLVSQIAFSAI<br>ELQKNGGDEYVEAALKVVKKAAEELGNSEEEALYWLVSQIAFSAIELQKNGGDEYVEAALK<br>VVKKAAEELGNSEEEALYWLVSQIAFSAIELQKNGGDEYVEAALKVVKKAAEELGNSEEEA<br>LYWLVSQIAFSAIELQKNGGDEYVEAALKVVKKAAEELGNSEDEALHLLKSQIAFSAHELEN<br>NGGEEYVKAAEKVEKKAAEELGNE
RPB_PKW1_R6-PKWx6	R6M4_b20	PKWPKWPKWPKWPKWPKW	PEEKLEEEVTQIARAAIYKQRNGGDEHVEKALKRVKRAAEELGNSEEEALEELVTQIARAAI<br>YKQRNGGDEYVEAALKVVKKAAEELGNSEEEALEELVTQIARAAIYKQRNGGDEYVEAAL<br>KVVKKAAEELGNSEEEALEELVTQIARAAIYKQRNGGDEYVEAALKVVKKAAEELGNSEEE<br>ALEELVTQIARAAIYKQRNGGDEYVEAALKVVKKAAEELGNSEDEAEEELKTQIARAAHYK<br>ENNGGEEYVKAAEKVEKKAAEELGNE
RPB_hy1-PLP$_2$PEP$_2$PLP$_2$	R6s23	PLPPLPPEPPEPPLPPLP	DEEREKLKEKLKEVLRRAKEAKKKGDKEKLIELAYEAAALAAWIIHKDSNDDEIVELAKEALK<br>LVLEAAKEAKKNGDKEKLIKLAYLAAAVAKWIIHTDGDDDEIVELAKEALKLVLEAAKEAKKN<br>GDKEKLIKLAKLAASVAKWIIHTDGDDDEIVELAKEALKLVLEAAKEAKKNGDKEKLIKLAKL<br>AASVAAWIIHTDGDDDEIVELAKEALKLVLEAAKEAKKNGDKEKLIKLAYLAAAVAAWIITTD<br>GDDEEIVELAKEALKLVKEAAEEAEKQGDEELREKLRYLSEAVREWIERNDGDD
	R6E2	PLPPLPPEPPEPPLPPLP	DEEREKLKEKLKEVLRRAKEAKKKGDKEKLIELAYEAAALAAWIIHKDSNDDEIVELAKEALK<br>LVLEAAKEAKKNGDKEKLIKLAYLAAAVAKWIIHTDGDDDEIVELAKEALKLVLEAAKEAKKN<br>GDKEKLIKLAKLAASVAAWIIHTDGDDDEIVELAKEALKLVLEAAKEAKKNGDKEKLIELAEL<br>AAAVAAWIIHTDGDDDEIVELAKEALKLVLEAAKEAKKNGDKEKLIKLAYLAAAVAAWIITTD<br>GDDEEIVELAKEALKLVKEAAEEAEKQGDEELREKLRYLSEAVREWIERNDGDD
RPB_hy2-IYP$_3$RYP	R6s59	IYPIYPIYPRYPRYPRYP	PEEEILVKAVNEASRITEKTKDEEKIKEALERILREAAERIRENGGNEEEAILVIAVNVASRITE<br>RVKNEEEIKKALEWILRVAAELIRENGGNEEEAISVIAINVASRITERVKNEEEIKKALEWILR<br>VAAELIRENGGNEEEAIVEIAINVASRITERVKNEEEIKKALEWILRVAAELIRENGGNEEEAI<br>VEIAINVASRITERVKNEEEIKKALEWILRVAAELIRENGGNEEEAILEIAVNVASRITKRVTNE<br>EEIKKAQHWIKHVAKKLIKENGGT
	R6s93	IYPIYPIYPRYPRYPRYP	PEEEILIKAVNEASRITEKTKDEEKIKEALERILREAAERIRENGGNEEEAILIIAVNVASRITER<br>VKNEEEIKKALEWILRVAAELIRENGGNEEDAISIIAINVASRITERVKNEEEIKKALEWILRVA<br>AELIRENGGNEEDAIVEIAINVASRITERVKNEEEIKKALEWILRVAAELIRENGGNEEDAIVEI<br>AINVASRITERVKNEEEIKKALEWILRVAAELIRENGGNEEDAILEIAVNVASRITKRVTNEEEI<br>KKAQHWIKHVAKKLIKENGGT
αZFC-high	endol_b2	PLPPLPPLPPLPPEDPEQPPKPPF	DEIVELAKEALKLVLEAAKEAKKNGDKEKLIKLAYLAAAVAAWIIHTDGDDDEIVELAKEALKL<br>VLEAAKEAKKNGDKEKLIKLAYLAAAVAAWIIHTDGDDDEIVELAKEALKLVLEAAKEAKKNG<br>DKEKLIKLAYLAAAVAAWIIHTDGDDDEIVELAKEALKLVLEAAKEAKKNGDKEKLIKLAYLAA<br>AVAAWIIHTDGDDDEIVELAKEALKLVLEAAKEAKKNGDKEKLIKLAYLAAAVAAWIIKTDGD<br>DDEIVELAKEALKLVLEAAKEAKKNGDKEKLIKLAYLAAAVAAWIIHTDGDDDEIVELAKEAL<br>KLVLEAAKEAKKNGDKEKLIKLAYLAASVAAWIITTDGDDEEIVELAKEALKLVKEAAEEAEK<br>QGDEELREKLRYLSEAVREWIERNDGDD
αZFC-low	endol_b7	PLPPLPPLPPLPPEDPEQPPKPPF	KEKKIEKAYRAAAEAAWKIHTDGDDDEIVEEAKEALKKVLEAAKEAKKNGDKEKLIKLAYLA<br>AAVAAWIIHTDGDDDEIVELAKEALKLVLEAAKEAKKNGDKEKLIKLAYLAAAVAAWIIHTDG<br>DDDEIVELAKEALKLVLEAAKEAKKNGDKEKLIKLAYLAAAVAAWIIITDGDDDEIVELAKEAL<br>KLVLEAAKEAKKNGDKEKLIKLAYLAAAVAAWIIHTDGDDDEIVELAKEALKLVLEAAKEAKK<br>NGDKEKLIKLAYLAASVAAWIITTDGDDEEIVELAKEALKLVKEAAEEAEKQGDEELREKLR<br>YLSEAVREWIERNDGDD
